# Insights Into the Prognostic Value and Immunological Role of NAAA in Pan-Cancer

**DOI:** 10.3389/fimmu.2021.812713

**Published:** 2022-01-06

**Authors:** Da Huang, Jiayu Shen, Lingyun Zhai, Huanhuan Chen, Jing Fei, Xiaoqing Zhu, Jianwei Zhou

**Affiliations:** ^1^ Department of Gynecology, The Second Affiliated Hospital, School of Medicine, Zhejiang University, Hangzhou, China; ^2^ Department of Obstetrics, The Second Affiliated Hospital, School of Medicine, Zhejiang University, Hangzhou, China

**Keywords:** N-Acylethanolamine Acid Amidase (*NAAA*), pan-cancer, prognosis, immune infiltration, tumor immunosuppressive status

## Abstract

N-Acylethanolamine Acid Amidase (*NAAA*) is an N-terminal cysteine hydrolase and plays a vital physiological role in inflammatory response. However, the roles of *NAAA* in tumor immunity are still unclear. By using a series of bioinformatics approaches, we study combined data from different databases, including the Cancer Genome Atlas, the Cancer Cell Line Encyclopedia, Genotype Tissue-Expression, cBioPortal, Human Protein Atlas, TIMER, and ImmuCellAI to investigate the role of *NAAA* expression in prognosis and tumor immunity response. We would like to reveal the potential correlations between *NAAA* expression and gene alterations, tumor mutational burden (TMB), microsatellite instability (MSI), DNA methylation, tumor microenvironment (TME), immune infiltration levels, and various immune-related genes across different cancers. The results show that *NAAA* displayed abnormal expression within most malignant tumors, and overexpression of *NAAA* was associated with the poor prognosis of tumor patients. Through gene set enrichment analysis (GSEA), we found that *NAAA* was significantly associated with cell cycle and immune regulation-related signaling pathways, such as in innate immune system, adaptive immune system, neutrophil degranulation, and Toll-like receptor signaling pathways (TLRs). Further, the expression of *NAAA* was also confirmed to be correlated with tumor microenvironment and diverse infiltration of immune cells, especially tumor-associated macrophage (TAM). In addition to this, we found that *NAAA* is co-expressed with genes encoding major histocompatibility complex (MHC), immune activation, immune suppression, chemokine, and chemokine receptors. Meanwhile, we demonstrate that *NAAA* expression was correlated with TMB in 4 cancers and with MSI in 10 cancers. Our study reveals that *NAAA* plays an important role in tumorigenesis and cancer immunity, which may be used to function as a prognostic biomarker and potential target for cancer immunotherapy.

## Introduction

As a worldwide threat to public health, malignant tumors not only bring endless suffering to patients and their families but also add huge economic burden to society. Early screening and subsequent surgical intervention have made an advanced progress in reducing the incidence and mortality of tumors, especially in colon and cervical cancer (1). However, the prognosis and survival rate of many types of cancer are still dissatisfied. Relapsing disease almost inevitably developed resistance to the initially sensitive drugs (2, 3). This implies several possible mechanisms are involved in the occurrence and development of multidrug resistance (MDR) in cancer (4). Tumor microenvironment may play a critical role in these potential mechanisms (5–7). Tumor microenvironment (TME) is a tumor-promoting setting that contains various cells, including innate and adaptive immune cells, cancer-associated fibroblasts (CAFs), tumor-associated endothelial cells (TECs), and extracellular matrix (8). Among all the cellular components, immune cells are the most important components. It is now known that interactions between tumor cells and the proximal immune cells can secrete cytokines and growth factors to promote tumor invasion, metastasis, and suppression of antitumor immunity (9, 10). Tumor immunotherapy, which is different from conventional chemotherapeutics, has witnessed dramatic advances in cancer treatment, particularly immune checkpoint blockade therapy (11, 12). The clinical success of immune checkpoint inhibitors (ICIs), such as anti-programmed cell death protein-1 (PD-1) or its ligand (PD-L1) and anti-cytotoxic T lymphocyte-associated protein 4 (CTLA-4), have been approved the standard of care in many types of malignancies (13–15). However, the overall response rates in many patients are still minimal when provided the same treatment. In addition to tumor cell-intrinsic factors, such as insufficient tumor antigenicity, disruption of interferon-γ signal pathway, and downregulation surface MHC-I level (16, 17), the TME plays a major role in immunosuppression and affects clinical outcomes of cancer patients. Therefore, there is an urgent need to search for new immune-related therapeutic targets in cancers.

N-Acylethanolamine acid amidase (*NAAA*) is a lysosomal enzyme that is primarily expressed in the adaptive and innate immune cells (18). Its known to promote inflammatory responses through regulating the deactivation of palmitoylethanolamide (PEA) (19, 20), an endogenous lipid mediator that ligates peroxisome proliferator-activated receptor α (PPAR-α) to diminish production of proinflammatory cytokines and achieves anti-inflammatory and analgesic effects (21–23). Therefore, multiple *NAAA* inhibitors have been developed to treat inflammatory-related diseases, including arthritis (24), lung inflammation (25), inflammatory bowel disease (26), and allergic contact dermatitis (27). Additionally, recent studies showed that *NAAA* inhibitors have an effect on the antitumor response. For example, Roberta et al. (27) showed that the *NAAA* inhibitors are able to significantly reduce proliferation and migration of bladder cancer cells.

Borrelli et al. (28) displayed *NAAA* inhibitors can induce colorectal cancer (CRC) cell cycle arrest in the S phase and reduce cell proliferation and migration. However, the potential role of *NAAA* in tumorigenesis and tumor progression remains fragmentary, and there are no bioinformatics analysis systematically exploring the relationship of *NAAA* expression between different types of human cancers.

Rapidly accumulating data from large‐scale cancer genomics studies, many studies have focused pan-cancer analysis to estimate the whole genome, frequently mutated genes and other common genomic characterization that are related to the occurrence and development of cancer (29–32). In this study, we examine the specific role and underlying mechanisms of *NAAA* in a pan-cancer dataset. On the one hand, we comprehensively deeply analyzed the association between *NAAA* expression and patient prognosis in 33 cancer types. In addition, we further assessed the expression of *NAAA* and its association with tumor-infiltrating immune cells. Our findings revealed the possible role of *NAAA* in tumorigenesis and progression of multiple cancers, suggesting that *NAAA* is a potential prognostic and immunotherapeutic biomarker.

## Materials and Methods

### *NAAA* Gene Data Collection and Processing

*NAAA* gene expression data and clinical information in tumor and corresponding normal samples were obtained from the Cancer Genome Atlas (TCGA) and the Genotype-Tissue Expression (GTEx) using UCSC Xena (https://xena.ucsc.edu/), an online tool for exploration of gene expression and clinical and phenotype data. The Cancer Cell Line Encyclopedia (CCLE) database was used to analyze *NAAA* expression in different cancer cell lines for a multidimensional investigation. The expression level of *NAAA* in 33 different human cancer tissues and 31 different normal tissues, as well as the corresponding 21 tumor cell lines, was systematically analyzed. The RNA sequencing data were Log_2_-transformed, and two sets of *t-tests* were conducted on these tumor types; the statistically significant difference was defined to be * *p* < 0.05; ** *p*< 0.01; *** *p* < 0.001. Data analysis was conducted using R software (Version 4.0.2), and the R package “ggpubr” was used to draw radar plots or boxplot. The compiled data were derived from 9,861 TCGA tumor tissues, 712 TCGA normal tissues, and 7,718 GTEx normal tissues, more details as seen in **Supplementary Table 1**.

### Immunohistochemistry Staining

Human Protein Atlas (HPA) (http://www.proteinatlas.org/), a landmark protein research database containing the protein expression of tumor tissues and normal tissues, was applied to explore *NAAA* expression at the protein level. IHC images of NAAA protein expression in normal and eight tumor tissues including breast invasive carcinoma (BRCA), ovarian cancer (OV), prostate adenocarcinoma (PRAD), stomach adenocarcinoma (STAD), colon adenocarcinoma (COAD), liver hepatocellular carcinoma (LIHC), uterine carcinosarcoma (UCS), and kidney renal papillary cell carcinoma (KIRP) were downloaded from the HPA and analyzed. The antibody used for IHC was CAB026135.

### *NAAA* Gene Expression and Survival Prognosis Analysis

Survival information of overall survival (OS), disease-specific survival (DSS), disease-free interval (DFI), and progression-free interval (PFI) were extracted from TCGA and to reveal the relationship between *NAAA* expression and patient prognosis. The median of *NAAA* expression in each tumor was used as cutoff value to divide patients into high- and low-expression subgroups. The survival data of each cancer type were assessed by Kaplan-Meier survival method and log-rank test. The survival curves were drawn using R packages “survminer” and “survival,” and *p <*0.05 was considered significant. Moreover, a univariate Cox model was used to evaluate the relationships between *NAAA* expression and various survival outcomes in a pan-cancer analysis, and a hazard ratio (HR) <1 was considered to mean that *NAAA* is a protection factor in cancer; otherwise, HR >1 means that *NAAA* is a risk factor in cancer. Data were visualized as forest plots (using the “forestplot” R package).

### *NAAA* Genetic Alteration Analysis

The cBioPortal database (www.cbioportal.org) was used to explore genomic alterations analyses for a specific gene (33). In this study, we applied the “Cancer Types Summary” and below “Cancer Type” button for visualizing genomic alterations of *NAAA* among 32 cancer types of TCGA. The results were presented with plotted bar plots, and the frequencies of *NAAA* copy number alterations and mutations in all TCGA tumors were observed. The HM450 methylation data of each tumor were also obtained from the cBioPortal database. The connection between the *NAAA* expression levels and methylation levels in its promoter region was analyzed for each cancer and visualized using the R package “ggpubr”.

### Tumor Mutation Burden and Microsatellite Instability

Tumor mutation burden (TMB) was defined as the total number of somatic coding mutations in a specific cancer, which were closely related to the effectiveness of immunotherapy across diverse types of human cancers. We downloaded somatic mutation data of all TCGA patients from the UCSC XENA database and calculated their TMB scores. Microsatellite instability (MSI) is a condition characterized by repetitive sequences of mono- and oligonucleotides (short tandem repeats) that reflect DNA mismatch repair (MMR) deficiency. Similarly, MSI is a marker for good response to immunotherapy. The microsatellite instability (MSI) data were obtained from a recent study (34). The telationship of *NAAA* expression with TMB or MSI was analyzed by utilizing Spearman’s correlation coefficient.

### Tumor Microenvironment or Infiltration of Immune Cells

Estimation of Stromal and Immune Cells in Malignant Tumor Tissues Using Expression Data (ESTIMATE) is a method to calculate stromal or immune scores, which represent the abundance of immune and stromal components, respectively. The higher the score the larger the ratio of the corresponding component in TME. ESTIMATE score, the sum of stromal and immune scores, represents the integrated proportion of both components in TME. The *NAAA* expression and ImmuneScore and StromalScore of each cancer were obtained *via* the “estimate” R package and Spearman’s correlation analysis. Immune cell infiltration correlation analysis was performed using two databases, including TIMER2 database (http://timer.cistrome.org) and ImmuCellAI database (http://bioinfo.life.hust.edu.cn/ImmuCellAI) to perform the correlation analysis. For each TCGA tumor type, patients were divided into two groups (high and low *NAAA* expression based on the median *NAAA* expression level) to compare the extent of immune cell infiltration.

### Gene Set Enrichment Analyses

Correlation analyses of NAAA with all genes were performed using TCGA data. Pearson’s correlation coefficients were calculated. Genes correlated with *NAAA* (p < 0.05) were selected for gene set enrichment analysis (GSEA). GSEA was performed using the R package “clusterProfiler” with the following parameters: nPerm = 1000, minGSSize = 10, maxGSSize = 1000, and *p*-value-Cutoff = 0.05. Gene sets from Reactome pathway database were selected for GSEA.

### Statistical Analysis

NAAA gene expression level differences in cancer tissues and normal tissues were estimated using t-tests. Survival analysis was analyzed by Kaplan–Meier method and compared using log-rank test, and the results were presented as hazard ratios, 95% CI, and *p*-values of log-rank tests. The correlation analysis between the two variables used Spearman’s or Pearson’s test. All the statistical analyses were conducted using R software (version 4.0.2). A *p*-value < 0.05 was considered statistically significant.

## Results

### NAAA Expression Analysis in Pan-Cancer

**Figure 1:**
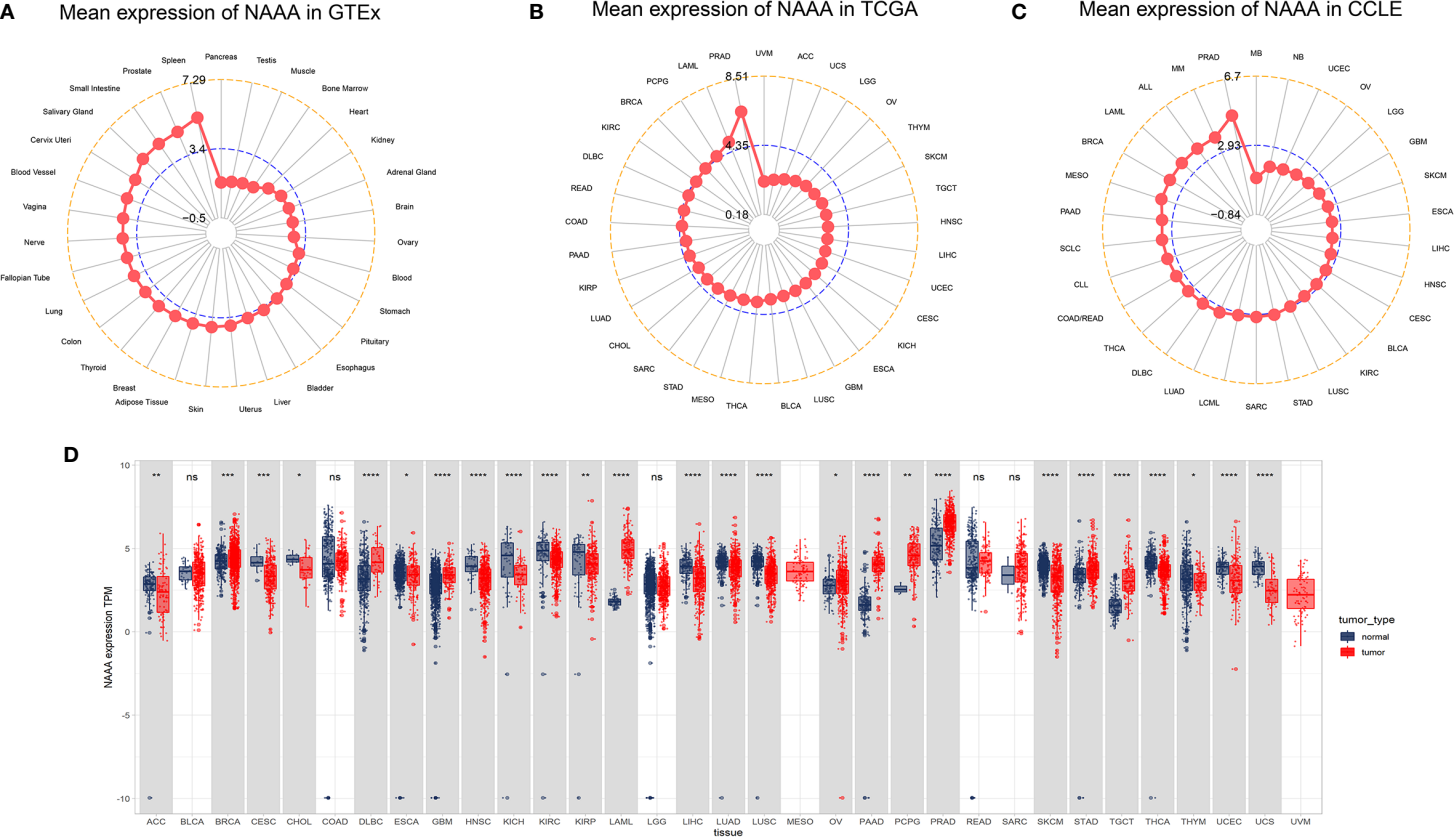
NAAA mRNA expression levels in pan-cancer. **(A)** NAAA expression levels in normal tissues from GTEx database. **(B)** NAAA expression levels in tumor tissues from TCGA database. **(C)** NAAA expression levels in tumor cell lines from CCLE database. **(D)** NAAA expression difference between tumor tissues from TCGA database and normal tissues from the GTEx database; ns, no significance; *p < 0.05, **p < 0.01, ***p < 0.001, and ****p < 0.0001.

First, we analyzed *NAAA mRNA* expression in normal tissues using the GTEx dataset. As shown in **Figure 1A**, the highest *NAAA* expression was observed in the spleen, prostate, and small intestine, while the lowest expression was detected in pancreas. For tumor tissues in TCGA, we found *NAAA* expression was the highest in prostate adenocarcinoma (PRAD) and lowest in uveal melanoma (UVM) (**Figure 1B**). In addition, we explored *NAAA* expression across different tumor cell lines in the CCLE database and found the gene was also the highest in PRAD (**Figure 1C**). To further compare *NAAA* expression between the tumor and normal tissues, we combined data from the GTEx and TCGA database to analyze the differences in *NAAA* expression. Results from databases revealed that *NAAA* was overexpressed in 10 of these cancers: breast invasive carcinoma (BRCA), lymphoid neoplasm, diffuse large B cell lymphoma (DLBC), glioblastoma multiforme (GBM), acute myeloid leukemia (LAML), ovarian cancer (OV), pancreatic adenocarcinoma (PAAD), pheochromocytoma and paraganglioma (PCPG), prostate adenocarcinoma (PRAD), stomach adenocarcinoma (STAD), testicular germ cell tumors (TGTC). In contrast, low *NAAA* expression was observed in 16 cancers: adrenocortical carcinoma (ACC), cervical squamous cell carcinoma (CESC), cholangiocarcinoma (CHOL), esophageal carcinoma (ESCA), head and neck squamous cell carcinoma (HNSC), kidney chromophobe (KICH), kidney renal clear cell carcinoma (KIRC), kidney renal papillary cell carcinoma (KIRP), liver hepatocellular carcinoma (LIHC), lung adenocarcinoma (LUAD), lung squamous cell carcinoma (LUSC), skin cutaneous melanoma (SKCM), thyroid carcinoma (THCA), thymoma (THYM), uterine corpus endometrial carcinoma (UCEC), uterine carcinosarcoma (UCS) (**Figure 1D**).

We also assessed the relationship of *NAAA mRNA expression* in different pathological stages of multiple cancer types and found that it was lower in higher stages in three cancer types, including BRCA, PAAD, and UCEC. In contrast, higher *NAAA* expression in higher stages was observed in BLCA, MESO, READ, and UCS. However, a contradictory conclusion was observed in THCA; *NAAA* expression was downregulated in stage II when compared to stage I but upregulated in stage III (**Supplementary Figure S1**).

For paired tumors and normal tissues in TCGA, NAAA was expressed at low levels in COAD, HNSC, KICH, KIRC, KIRP, LIHC, LUAD, LUSC, READ, and THCA, while *NAAA* high expression was only observed in PRAD (**Supplementary Figure S2**).

**Figure 2:**
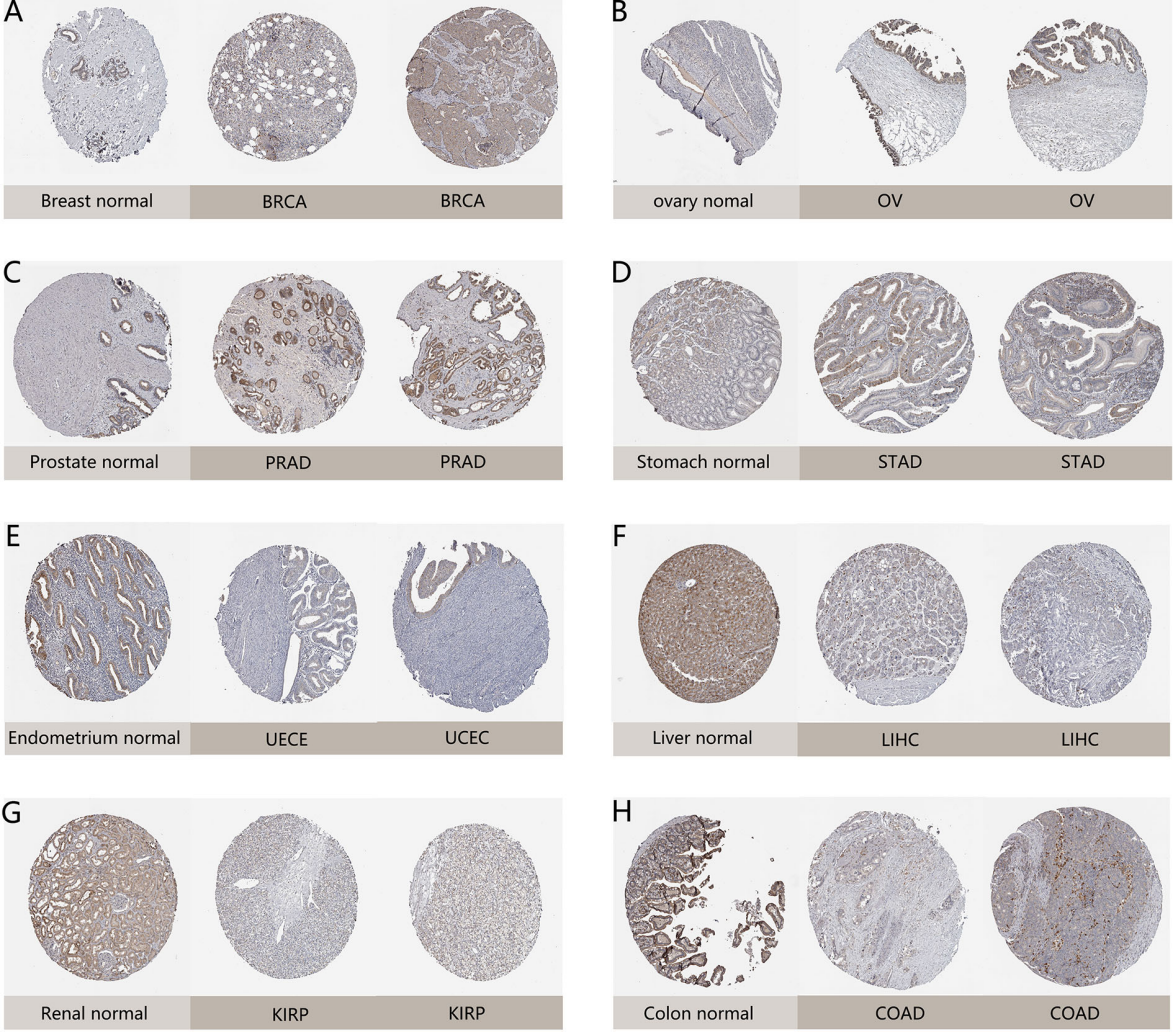
Representative immunohistochemical staining (IHC) in various normal (left) and tumor (right) tissues. The protein expression of NAAA in **(A)** lobular breast carcinoma, BRCA; **(B)** ovarian serous cystadenocarcinoma, OV; **(C)** prostate adenocarcinoma, PRAD; **(D)** stomach adenocarcinoma, STAD; **(E)** endometrial adenocarcinoma, UCEC; **(F)** liver hepatocellular carcinoma, LIHC; **(G)** kidney renal papillary cell carcinoma, KIRP; **(H)** colon adenocarcinoma, COAD.

Moreover, we further evaluated *NAAA* expression between normal and tumor tissues at protein level from HPA database. As shown in **Figure 2**, compared to weak IHC staining in normal stomach and breast, a much stronger staining of NAAA was detected in BRCA and STAD tissues. Normal ovary tissue samples had negative NAAA staining, while tumor tissues had weak staining. Normal prostate, liver, renal, colon, and endometrium had medium NAAA staining, while PRAD tissues had strong staining; and UCES, LIHC, KIRP, and COAD tissues had weak NAAA IHC staining. The data analysis results from the two databases were consistent with each other.

### Prognostic Value of *NAAA*

**Figure 3:**
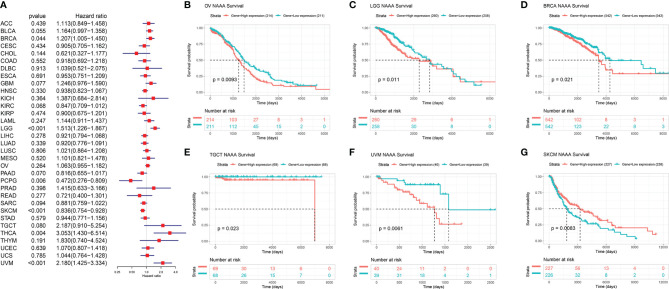
Relationship of NAAA expression with patient Overall Survival (OS). **(A)** Forest map shows the univariate Cox regression analysis results for NAAA in TCGA pan-cancer samples. **(B–G)** Kaplan–Meier OS curves of NAAA expression in the six most significantly associated tumors.

**Figure 4:**
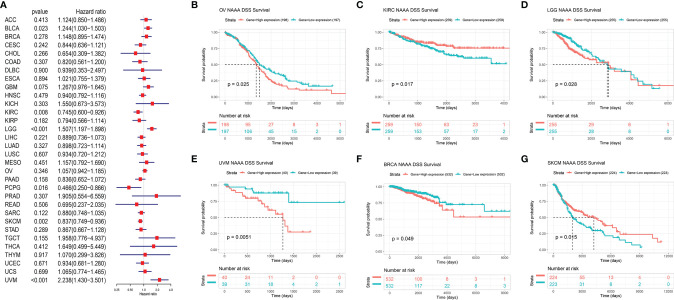
Relationship of NAAA expression with patient Disease-Specific Survival (DSS). **(A)** Forest map shows the univariate Cox regression analysis results for NAAA in TCGA pan-cancer samples. **(B–G)** Kaplan–Meier DSS curves of NAAA expression in the six most significantly associated tumors.

**Figure 5:**
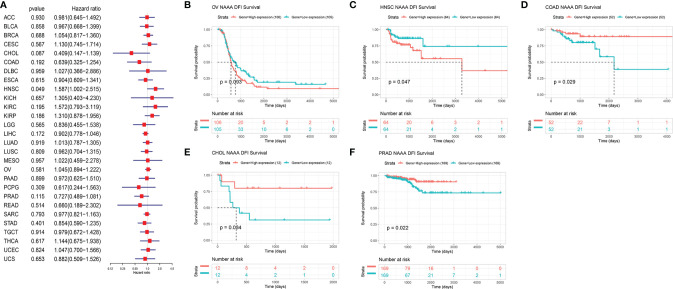
Relationship of NAAA expression with patient Disease-Free Interval (DFI). **(A)** Forest map shows the univariate Cox regression analysis results for NAAA in TCGA pan-cancer samples. **(B–F)** Kaplan–Meier DFI curves of NAAA expression in the five most significantly associated tumors.

**Figure 6:**
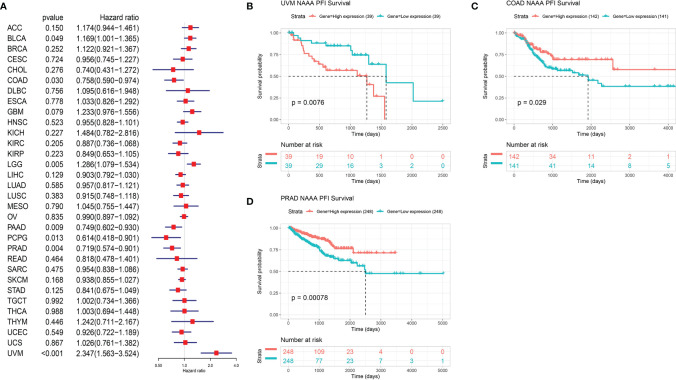
Relationship of NAAA expression with patient Progression-Free Interval (PFI). **(A)** Forest map shows the univariate Cox regression analysis results for NAAA in TCGA pan-cancer samples. **(B–D)** Kaplan–Meier PFI curves of NAAA expression in the three most significantly associated tumors.

Next, we investigated the associations between *NAAA* expression and cancer survival outcomes, including OS, DSS, DFI, and PFI. As shown in **Figure 3A**, the results of univariate Cox regression analysis suggested that *NAAA* was a risk factor for OS in BRCA, LGG, THCA, and UVM patients, while it was a protective factor in PCPG and SKCM patients. The Kaplan-Meier OS analysis showed that high expression of *NAAA* was significantly correlated with poor prognosis of patients in BRCA, LGG, OV, UVM, TCGT, but better prognosis was found in SKCM **Figures 3B**–**G**. Then, Cox regression analysis of DSS identified that *NAAA* was a risk factor in BLCA, LGG, and UVM. However, it acted as a protective factor in KIRC, PCPG, and SKCM, as seen in **Figure 4A**. KM analysis showed that patients with higher *NAAA* expression had poorer DSS than those with lower *NAAA* expression in OV, LGG, UVM, and BRCA. Patients with increased *NAAA* levels showed superior DSS to those with decreased *NAAA* levels in KIRC and SKCM **Figures 4B**–**G**. Furthermore, Cox regression analysis of DFI analyses showed *NAAA* was a risk factor in HNSC (**Figure 5A**). And KM analysis showed that patients with higher *NAAA* expression had poorer DFI than those with lower *NAAA* expression in OV and HNSC, but contrasting result was shown in COAD, CHOL, and PRAD (**Figure 5B**–**F**). Finally, Cox regression analysis of PFI revealed that *NAAA* acts as risk factor for patients with BLCA, LGG, and UVM, while as a protective factor in COAD, PAAD, PCPG, and PRAD (**Figure 6A**). And KM analysis showed that patients with higher *NAAA* expression had poorer PFI than those with lower *NAAA* expression in UVM and reversely in COAD and PRAD, as seen in **Figures 6B**–**D**.

### Genetic Alteration of *NAAA*

**Figure 7:**
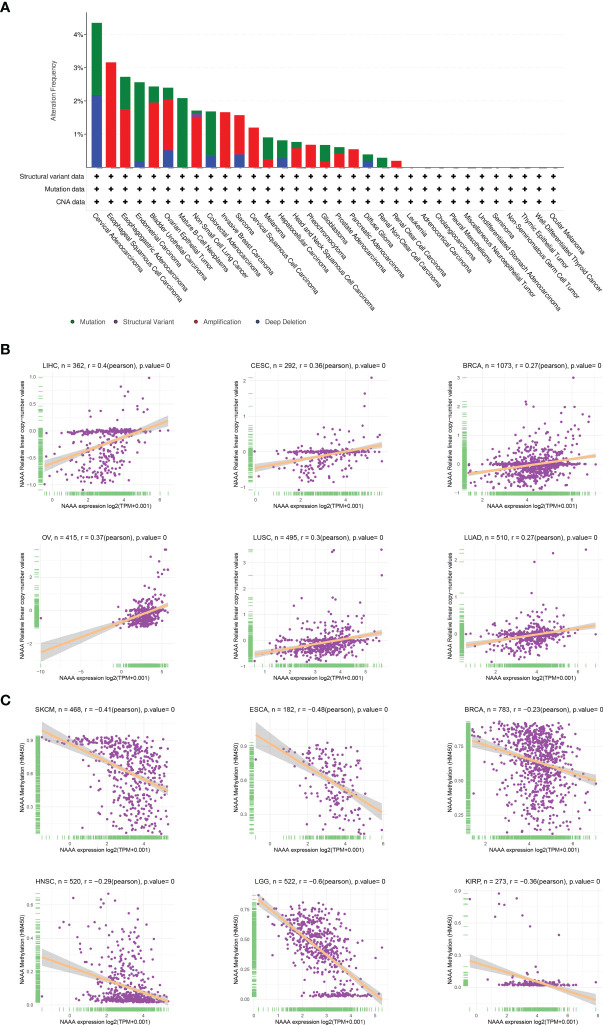
Relationship of NAAA expression with gene alterations. **(A)** The genetic alteration type and frequency of NAAA in various cancers. **(B)** Correlation between NAAA expression and gene promoter methylation in liver hepatocellular carcinoma (LIHC), cervical squamous cell carcinoma (CESC), breast invasive carcinoma (BRCA), ovarian cancer (OV), lung squamous cell carcinoma (LUSC), lung adenocarcinoma (LUAD). **(C)** Correlation between NAAA expression and copy number variation in skin cutaneous melanoma (SKCM), esophageal carcinoma (ESCA), breast invasive carcinoma (BRCA), head and neck squamous cell carcinoma (HNSC), brain lower grade glioma (LGG), kidney renal papillary cell carcinoma (KIRP).

DNA methylation and genetic alteration are closely associated with tumorigenesis and progression. We firstly assessed the *NAAA* alteration frequency and mutation count in cancer patients using cBioPortal database. Among all cancers, the highest alteration frequency of NAAA (> 4%) appears for patients in cervical cancer, with “mutation and deep deletion” as the primary types. The “amplification” type was the primary type in ESCA, CESC, pheochromocytoma, PAAD, and renal clear cell carcinoma, which show an alteration frequency of ~4% (**Figure 7A**). DNA methylation, as one of well-studied epigenetic modifications, often leads to the silencing or inactivation of tumor suppressor genes and thus contributes to initiation and proliferation of cancers (35, 36). We further calculated the levels of correlation between *NAAA* promoter methylation and identified significant correlations between gene expression and methylation in 22 tumors (**Table 1**). In ACC, BLCA, BRCA, CESC, COAD, ESCA, GBM, HNSC, KIRC, KIRP, LGG, LUAD, LUSC, PCPG, PRAD, SARC, SKCM, STAD, THCA, THYM, UCEC, and UVM, there were negative correlations between *NAAA* expression and promoter methylation levels. The six strongest negative correlations (BRCA, ESCA, HNSC, KIRP, LGG, and SKCM) are presented in **Figure 7B**. Further, the correlation analysis between NAAA and copy number variation (CNV), another commonly seen genetic mutation, was also performed in pan-cancer (37). The results revealed that the mRNA expressions of *NAAA* was mainly positively correlated with CNV (**Table 1**). In BLCA, BRCA, CESC, COAD, ESCA, GBM, HNSC, KIRC, KIRP, LGG, LIHC, LUAD, LUSC, MESO, OV, PAAD, SARC, STAD, TGCT, THCA, UCEC, and UCS, there were positive correlations between *NAAA* expression and promoter methylation levels; the six strongest positive correlations (BRCA, CESC, LIHC, LUSC, LUAD, and OV) are presented in **Figure 7C**.

**Table 1:** Correlation of NAAA expression with methylation and copy number variation analysis.

	Methylation		Copy Number Variation	
Cancer	P-value	Correlation	P-value	Correlation
ACC	0.004550805	-0.320016321	0.256269616	0.132736045
BLCA	0.001982189	-0.15287683	0.113120848	0.268654104
BRCA	9.49E-11	-0.228683432	0.247664955	0.123113994
CESC	4.98E-05	-0.229731882	0.147046089	0.214817014
CHOL	0.052823966	-0.325366583	0.339215394	-0.119501599
COAD	2.38E-06	-0.278618603	0.772627112	0.022663371
READ	0.114475676	-0.166604849	0.104984678	0.128235935
DLBC	0.070736134	-0.265993722	0.10437345	0.073529059
ESCA	9.08E-12	-0.477789945	0.310050494	0.053278607
GBM	0.000236953	-0.447361203	0.60609446	0.047953161
HNSC	7.92E-12	-0.294005266	0.613365118	0.05772056
KICH	0.713369427	0.046072026	0.000738211	0.167451302
KIRC	0.003338785	-0.164355696	1.91E-19	0.270474617
KIRP	8.48E-10	-0.360445652	1.56E-10	0.363201226
LAML	0.688192779	-0.031000299	0.005164068	0.165532406
LGG	7.36E-52	-0.597656118	0.009423023	0.193038924
LIHC	0.901785221	-0.006428573	0.023482029	0.187440243
LUAD	2.80E-08	-0.256945447	0.000246882	0.161324585
LUSC	1.94E-06	-0.245144142	0.002464717	0.132250724
MESO	0.646911855	-0.049798595	0.020267399	0.137215962
OV	0.237014254	0.439106177	5.18E-05	0.179081071
PAAD	0.513426187	-0.049160651	1.33E-15	0.40342564
PCPG	4.08E-09	-0.418618998	5.91E-10	0.269783408
PRAD	1.06E-05	-0.196326647	1.03E-11	0.299437991
SARC	0.000289227	-0.222195214	3.69E-05	0.427174029
SKCM	4.83E-20	-0.406430696	5.69E-15	0.370779953
STAD	7.87E-05	-0.203529074	6.02E-09	0.419789451
TGCT	0.956449786	-0.0047087	0.003997335	0.18001532
THCA	0.00169045	-0.138973275	6.32E-05	0.195780398
THYM	0.000162964	-0.33892476	0.004734873	0.244428823
UCEC	0.04466379	-0.151118898	0.036914991	0.094202191
UCS	0.271786752	-0.148036333	0.002403654	0.224892979
UVM	0.000529889	-0.381113234	0.004840688	0.371310018

### GSEA of *NAAA* Analysis

**Figure 8:**
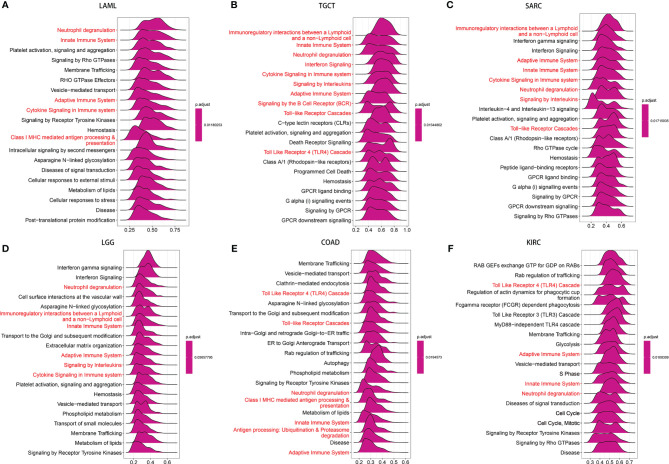
GSEA of NAAA in pan-cancer. **(A–F)** TOP20 GSEA terms in indicated tumor types. Red indicates immune regulation-related terms.

To investigate the biological function of *NAAA* expression in different tumor tissues, we evaluated the pathway through which *NAAA* may involve using GSEA in 33 tumor types from TCGA. The results revealed that *NAAA* participates in immune regulation-related pathways in pan-cancer, especially for the adaptive immune system, the innate immune system, immunoregulatory interactions between lymphoid, neutrophil degranulation, and Toll-like receptor signaling pathways (TLRs) (**Figures 8A**–**F**). These results suggest that *NAAA* plays an important role in regulating the inflammatory response and the tumor immune microenvironment.

### Tumor Microenvironment Analysis

**Figure 9:**
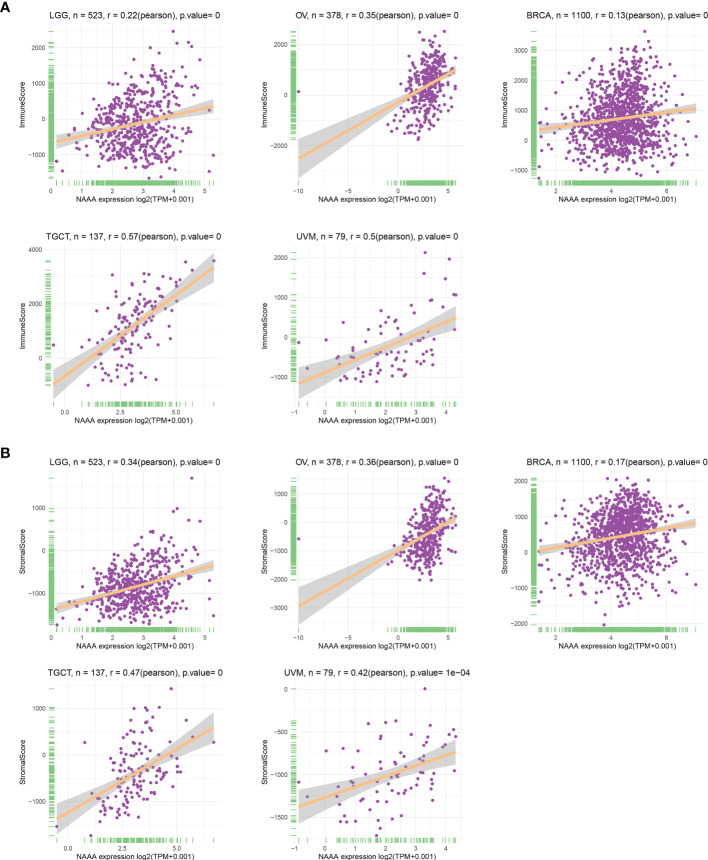
Relationship of NAAA expression with stromal score and immune score in five cancers. NAAA expression has a significantly positive correlation with the StromalScore **(A)** and ImmuneScore **(B)** in LGG, OV, BRCA, TGCT, UVM. BRCA, breast invasive carcinoma; LGG, brain lower grade glioma; UVM, uveal melanoma; OV, ovarian cancer; TGTC, testicular germ cell tumors.

As the survival places of tumor cells, the tumor microenvironment plays a crucial role in multidrug resistance and contribute to the development of cancer cell progression and metastasis. Therefore, we investigated the correlations of *NAAA* expression and the composition of tumor microenvironment by adopting ESTIMATE algorithm to calculate the immune and stromal scores, respectively. As shown in **Table 2**, *NAAA* expression was positively correlated with the immune scores in DLBC, TGCT, SARC, UVM, LUSC, LAML, GBM, SKCM, LUAD, MESO, OV, HNSC, BLCA, ESCA, KIRC, LGG, STAD, THCA, CESC, PAAD, LIHC, BRCA and negatively correlated with the Immune Score in PRAD. In addition, the expression of *NAAA* was positively correlated with the stromal scores in 11 cancer types, including SARC, GBM, LAML, TGCT, SKCM, LUSC, THYM, UVM, LUAD, MESO, DLBC, OV, LGG, HNSC, THCA, BLCA, PAAD, LIHC, KIRC, STAD, ESCA, BRCA, KIRP, and CESC, while negatively correlated in PARD and PCPG (**Table 2**). The five cancer types of poor OS (according to Kaplan-Meier and Cox survival analyses) with a positive correlation between TME and *NAAA* expression are presented in **Figure 9**.

**Table 2:** Correlation of NAAA expression with ImmuneScore and StromalScore analysis.

Cancer	ImmuneScore	StromalScore	EstimateScore
ACC	0.603486902	0.574888796	0.5756947
BLCA	3.89E-11	1.91E-11	9.62E-13
BRCA	2.79E-05	1.27E-08	4.09E-08
CESC	0.002592198	0.013634842	0.001763508
CHOL	0.166072766	0.063339129	0.095905818
COAD	0.115743977	0.253871171	0.155610566
READ	0.060145104	0.105801061	0.067339672
DLBC	5.99E-10	0.010150231	3.74E-07
ESCA	0.00023586	0.02919442	0.001548439
GBM	9.89E-11	1.34E-13	8.32E-13
HNSC	1.60E-15	4.17E-14	1.13E-18
KICH	0.982955885	0.371067144	0.693924553
KIRC	2.07E-11	9.84E-11	1.23E-13
KIRP	0.877319124	0.004591607	0.198539386
LAML	9.10E-10	1.03E-10	1.39E-11
LGG	3.09E-07	6.33E-16	1.05E-10
LIHC	0.006673596	4.64E-09	9.32E-06
LUAD	4.65E-25	1.33E-19	2.86E-26
LUSC	5.40E-31	4.17E-26	1.92E-32
MESO	0.000521863	0.000289806	4.30E-05
OV	1.34E-12	2.87E-13	1.79E-15
PAAD	0.024069576	1.09E-05	0.000514816
PCPG	0.200729171	0.011658308	0.043724062
PRAD	1.22E-06	0.007829048	2.95E-05
SARC	1.31E-19	2.13E-24	4.95E-25
SKCM	3.79E-25	1.64E-26	2.98E-30
STAD	2.35E-05	0.000511029	2.44E-05
TGCT	3.14E-13	8.32E-09	4.57E-19
THCA	1.16E-06	4.84E-14	2.79E-10
THYM	0.695421876	1.38E-06	0.004258205
UCEC	0.415833445	0.573729094	0.427772794
UCS	0.05079601	0.08374128	0.034629708
UVM	2.24E-06	0.000129013	2.61E-06

### Immune Cell Infiltration Analysis

**Figure 10:**
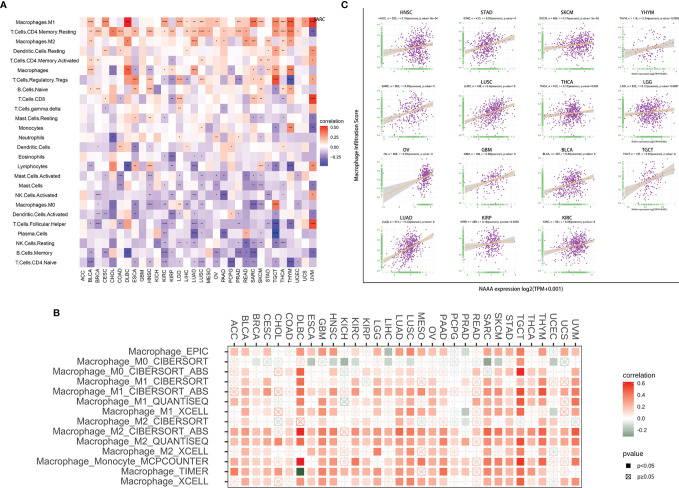
Relationship of NAAA expression with Immune cell infiltration analysis. **(A)** The relationship between NAAA expression levels and the levels of infiltration of 26 immune-related cells. **(B)** The correlation between NAAA expression and TAM infiltration levels by using TIMER2 database. **(C)** The correlation between NAAA expression and TAM infiltration by using ImmuCellAI database.

To investigate the relationship of immune cell infiltration and *NAAA* expression at the pan-cancer level, we downloaded immune cell infiltration data from various database to conducted correlation analyses. According to published data, we evaluated the 26 immune cells using “CIBERSOFT” algorithm. Overall, *NAAA* expression was positively correlated with infiltrating levels of multiple immune cells including macrophages, CD4+ T cells, dendritic cells, and Tregs, whereas it was negatively correlated with memory B cells, naïve T cells, dendritic cells, plasma cells, and NK cells. Interestingly, the expression of *NAAA* had different relationships with different subgroups of infiltrating macrophages, which positively correlated with the levels of infiltrating M1 and M2 macrophages but negatively associated with M0 macrophages (**Figure 10A**). Furthermore, the results of the TIMER2 database also proved that *NAAA* expression positively correlated with the infiltration level of TAMs (**Figure 10B**). In line with above results, data from the ImmuCellAI database obtained the same result that *NAAA* expression was positively correlated with the level of TAMs in pan-cancer (**Figure 10C**). The results of immune cell infiltration data from the three different sources were consistent. These results indicate that *NAAA* may contribute to increase the infiltration levels of TAMs, which may explain its tumorigenicity role in most tumor types.

### Tumor Mutational Burden and Microsatellite Instability Analysis

**Figure 11:**
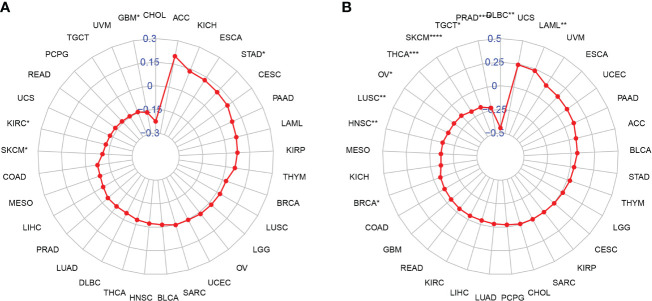
Relationship of NAAA expression and tumor mutational burden (TMB), microsatellite instability (MSI). **(A)** Radar map illustrating the relationship between NAAA expression and TMB. **(B)** Radar map illustrating the relationship between NAAA expression and MSI. The red lines represent correlation coefficients, and blue values represent ranges. Spearman correlation test, *p < 0.05, **p < 0.01, ***p < 0.001, and ****p < 0.0001.

TMB and MSI have been well-known to predict immune therapy response across different tumor types. Many studies revealed that patients with high TMB/MSI-H increased response rates and showed better outcomes to treatment with immunotherapy. We then evaluated their respective relationships with *NAAA* expression in pan-cancer, as shown in **Figure 11**. The correlation between *NAAA* expression and TMB achieved significance (P< 0.05) in four types of cancer. In general, *NAAA* expression was negatively correlated with TBM in GBM, KIRC, and SKCM while positively correlated with TMB only in STAD (**Figure 11A**). Similarly, we further found that the expression of *NAAA* was negatively related to the MSI of nine cancers, including BRCA, DLBC, HNSC, LUSC, OV, PRAD, SKCM, TGCT, and THCA, but had a positive correlation with MSI only in LAML (**Figure 11B**).

### Immune-Related Genes Analyses

**Figure 12:**
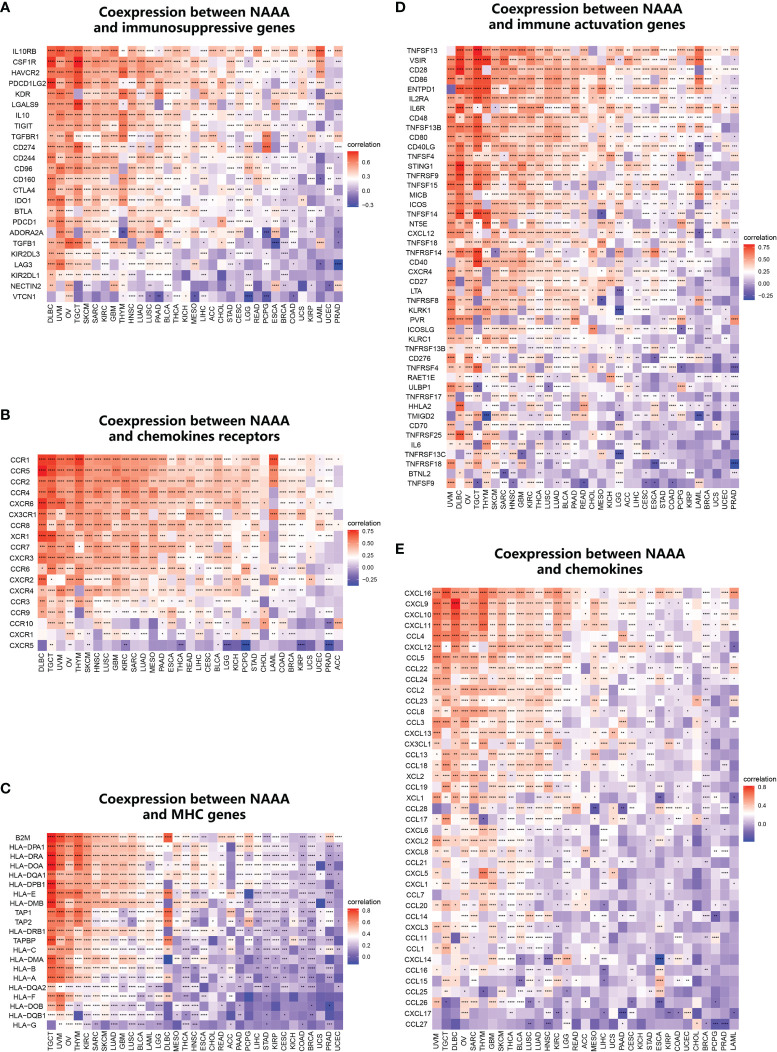
Co-expression of NAAA with immune-associated genes. Co-expression between NAAA and **(A)** MHC genes, **(B)** immune activated genes, **(C)** immunosuppressive genes, **(D)** chemokines, **(E)** chemokine receptors. *p < 0.05, **p < 0.01, ***p < 0.001, and ****p < 0.0001. Genes encoding MHC, immune activation, immune suppression, chemokine, and chemokine receptor proteins were analyzed.

Furthermore, we conducted gene co-expression analyses to explore the relationships between *NAAA* expression and immune-related genes in different cancer types. Genes encoding MHC, immune activation, immune suppression, chemokine, and chemokine receptor proteins were analyzed (**Figure 12**). According to the results, strong correlations were found between NAAA and most of the immune-related genes in specific cancer types, such as OV, UVM, DLBC, THYM, and TGCT. In detail, chemokine receptors such as CCR1, CCR5, and CCR2 and chemokines such as CXCL16, CXCL9, and CXCL10 were positively correlated with *NAAA* expression in various tumors. MHC genes had co-expression with *NAAA* in almost all cancer types, particularly in UVM, OV, THYM, TGCT, KIRC, SARC, and SKCM. In addition, immune activation genes and immunosuppressive genes were also closely correlated with *NAAA* expression in TCGA pan-cancer. To conclude, these results show that the expression of *NAAA* closely correlates with the biological function of various immune-related genes and cytokines.

## Discussion

As an important hallmark of cancer, inflammation contributes to the development of cancer and promotes tumor progression (38). About 20% of human cancers are closely associated with chronic inflammation. A number of soluble and cellular pro-inflammatory mediators (for example, chemokines, cytokines, and prostaglandins) are found in TME, where they play pivotal roles in tumor initiation, progression, and metastasis (39–41). NAAA, a lysosomal enzyme, is abundantly distributed in macrophages (42). It is already known that the inhibition of *NAAA* expression can effectively control inflammation by restoring endogenous PEA capacity *via* PPAR-α. In spite of the important roles of *NAAA* in the immune system, the correlation of *NAAA* function in immuno-oncology is still unknown. Here, we conducted a pan-cancer bioinformatics analysis of the expression profile and prognostic value of *NAAA* and explore its potential role in tumor immunology (43).

We first assessed the expression and prognostic significance of *NAAA* in pan-cancer using the GTEx and TCGA datasets. The results showed that compared to paracancerous and normal tissues, *NAAA* gene mRNA was highly expressed in 10 types of cancers, namely, BRCA, DLBC, GBM, LAML, OV, PAAD, PCPG, PRAD, STAD, and TGTC, whereas low expression was observed in ACC, CESC, CHOL, ESCA, HNSC, KICH, KIRC, KIRP, LIHC, LUAD, LUSC, SKCM, THCA, THYM, UCEC, and UCS. IHC analysis from the HPA was consistent with the NAAA mRNA expression discrepancy and complemented our results. Previous studies only revealed there is a relationship between NAAA expression and tumor cell progression in bladder, prostate, and colon cancer cells (28, 44, 45). Unfortunately, *NAAA* has not been largely studied in the cancer field. In this study, based on Kaplan-Meier and univariate Cox regression analysis, we also found that upregulated expression of *NAAA* was associated with poor prognosis, especially in patients with OV, LGG, UVM and BRCA. However, the high expression of *NAAA* related to a better OS prognosis in patients with SKCM, which means the function of *NAAA* was orientated more like a protective role in this specific cancer. Meanwhile, previous studies reported that the expression levels of *NAAA* mRNA were higher in non-aggressive prostate cancer than aggressive cancer and are potentially useful as a tissue biomarker related to cancer aggressiveness (46). These findings clearly suggest that *NAAA* is a potential biomarker to predict the prognosis of tumor patients.

Another important finding of this study is that *NAAA* plays an essential role in cancer immunity. Over the past years, more and more studies have illustrated that the immune status of tumors closely depended on the composition and infiltrating concentration of cells in their corresponding environment (47–49). ESTIMATE algorithm was proven to be convenient and fast to predict tumor purity, which reflects the features of TME, and has proven to be a prognostic factor in human malignancies, especially in colon cancer patients (50). Using the TCGA cohort, we found that *NAAA* was significantly positively correlated with the immune component of TME in 22 cancers, including DLBC, TGCT, SARC, UVM, LUSC, LAML, GBM, SKCM, LUAD, MESO, OV, HNSC, BLCA, ESCA, KIRC, LGG, STAD, THCA, CESC, PAAD, LIHC, and BRCA, and negatively correlated with the stromal component of TME in 2 cancers, including PCPG and PRAD. In addition, GSEA analysis indicates that *NAAA* was significantly associated with immune-related pathways, especially Toll-like receptor signaling pathways (TLRs). TLRs are a well-characterized family of Pattern recognition receptors (PRRs), and the latter are a significant component of the innate immune system. Many studies have reported that TLRs could stimulate several downstream signaling pathways and thus are involved in the pathogenesis of immune diseases and cancer (51, 52). The current findings suggested that *NAAA* is strongly associated with regulating innate immunity in some cancers by activating Toll-like receptor pathways. In addition, we found that *NAAA* was positively correlated with infiltrating levels of multiple immune cells including macrophages, CD4+ T cells, dendritic cells, and Tregs *via* using TIMER2 database. More importantly, immune cell infiltration data from ImmuCellAI database and a published article, we observed that *NAAA* expression was significantly correlated with tumor-associated macrophages (TAMs). TAMs are the most abundant population of tumor-infiltrating immune cells in TME and generally polarize into two functionally contrasting subtypes, namely, classically activated M1 and alternatively activated M2 subtypes (53). Compared to the former, tumor-infiltrating M2 macrophages are closely correlated with worse clinical prognosis in many kinds of malignant tumors (54). Increasing preclinical and clinical studies suggest that TAMs-targeting strategies could decrease the number of suppressive macrophages within tumors, which can be leveraged to potentiate the efficacy of immune checkpoint blockade (55). Based on these data, we propose that *NAAA* may have direct or indirect effects on macrophage polarization and subsequent induction of an immunosuppressive response. Furthermore, we further found that *NAAA* is co-expressed with genes encoding MHC, immune activation, immune suppression, chemokines, and chemokine receptor proteins. All these findings suggest that *NAAA* expression is closely correlated with immune infiltration of tumor cells and therefore affects patient prognosis.

TMB reflects the overall neoantigen burden within a tumor and thus is closely related to the efficacy of immunotherapy. Previous research has shown that high TMB correlates with better clinical outcomes from ICIs in patients with melanoma (56, 57), head and neck cancer (NSCLC) (58, 59), and urothelial carcinoma (60, 61). Furthermore, TMB may be useful as prognostic and predictive biomarker for immunotherapy response in human cancer. MSI also is a key biological marker of ICI response. The Food and Drug Administration (FDA) has approved microsatellite instability-high (MSI-H) status or deficient mismatch repair (dMMR) as predictive biomarkers for guiding the clinical application of ICIs in certain cancers (62). In the present study, we demonstrated that *NAAA* expression is correlated with TMB in four cancer types and with MSI in nine cancer types. Therefore, *NAAA* may serve as a potential predictor for the efficacy of immunotherapy in these types of cancers.

However, we must acknowledge some limitations in the current study. First, although we integrated and analyzed information across multiple databases, these datasets are grouped together without assessing heterogeneity, which may reduce the reliability of our findings. Second, the conclusions of this study are all drawn through bioinformatic analysis. There were no *in vitro/in vivo* experiments to verify the results. Previous studies reported multiple variants of *NAAA* mRNA found in various human cells and suggested that the proteins from some variants are catalytically inactive (63). Next, we will complement relative experiments to clarify the mechanism of *NAAA* in different types of cancer at both cellular and molecular levels. Third, despite the finding that *NAAA* expression was correlated with immune cell infiltration and patient survival in cancers, we are unable to confirm whether *NAAA* may affect patient survival *via* immune pathway at present.

In summary, our study systematically demonstrated the expression and prognostic value of *NAAA* across a series of cancers. The aberrant expression of *NAAA* is related to the poor prognosis in multiple cancer types and correlated with immune infiltration in TME, particularly with TAMs. In addition, *NAAA* expression was associated with TMB and MSI in multiple cancer types, which indicates *NAAA* associated with current predictors for the efficacy of ICIs. Nevertheless, these results were based on diverse data analysis, and future prospective and experimental studies are needed to demonstrate the specific role of *NAAA* in malignancies.

## Data Availability Statement

The datasets presented in this study can be found in online repositories. The names of the repository/repositories and accession number(s) can be found in the article/**Supplementary Material**.

## Author Contributions

DH, JS, and LZ participated in the study design and wrote the manuscript. DH and HC performed the data analysis. JF, XZ and JZ helped the revision. All authors contributed to the article and approved the submitted version.

## Funding

This study was supported by the National Nature Science Foundation of China (grant numbers 81902633) and the Natural Science Foundation of Zhejiang Province (grant number Y20H160278).

## Conflict of Interest

The authors declare that the research was conducted in the absence of any commercial or financial relationships that could be construed as a potential conflict of interest.

## Publisher’s Note

All claims expressed in this article are solely those of the authors and do not necessarily represent those of their affiliated organizations, or those of the publisher, the editors and the reviewers. Any product that may be evaluated in this article, or claim that may be made by its manufacturer, is not guaranteed or endorsed by the publisher.
