# Supplementary material for: Insights Into the Prognostic Value and Immunological Role of NAAA in Pan-Cancer
Source: Front Immunol. 2022 Jan 6;12:812713. doi: 10.3389/fimmu.2021.812713 (PMC8772335; doi:10.3389/fimmu.2021.812713)

Supplementary Figure 1: Pan-cancer NAAA expression in different stages. (A-H) Pan-cancer differential expression of NAAA in WHO stages in indicate tumor types from TCGA database.ns, not significant. **p* < 0.05 and ***p* < 0.01.


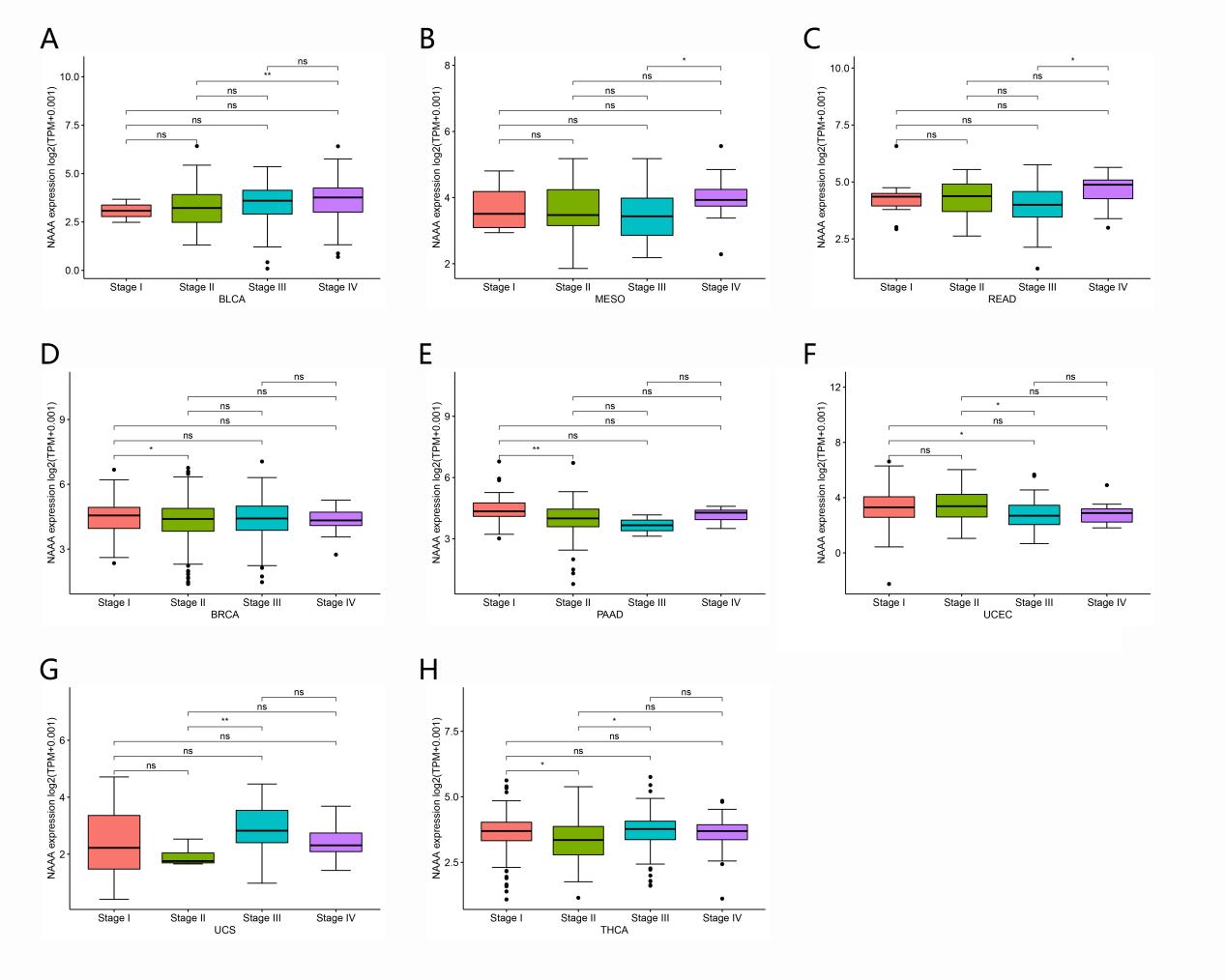


Supplementary Figure 2: Pan-cancer paired NAAA expression. (A-K) Pan-cancer NAAA expression in paired tumor and adjacent normal tissues in indicated tumor types from TCGA database. **p < 0.01 and ****p < 0.0001.


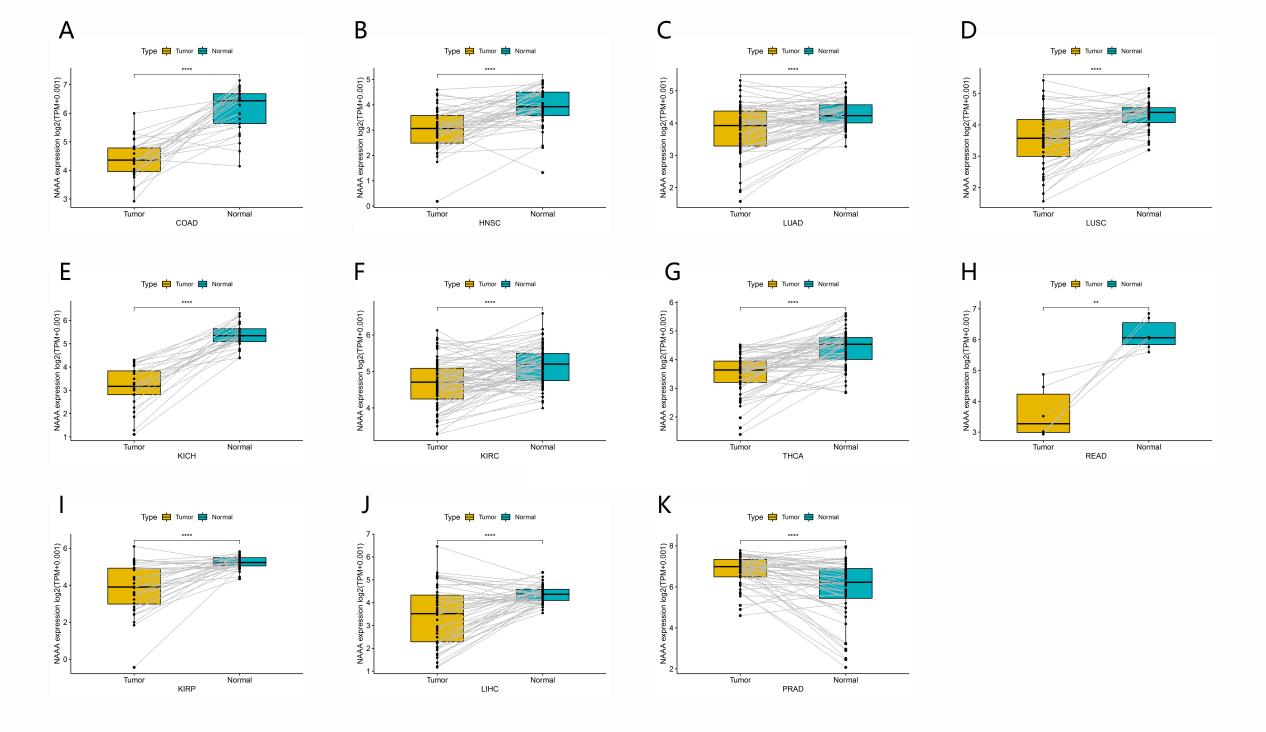

Supplement: Supplementary file 1 [file DataSheet_1.docx]
